# Intellectual disability and structural defects of the CaV2.1 channel in episodic ataxia type 2: correlation using an AI prediction model

**DOI:** 10.1007/s00415-026-13731-2

**Published:** 2026-03-04

**Authors:** Hyo-Jung Kim, Jin-Ok Lee, Sejoon Lee, Seoyeon Kim, Ji-Soo Kim

**Affiliations:** 1 Biomedical Research Institute, Seoul National University Bundang Hospital, Seongnam, South Korea; 2 Department of Health Science and Technology, Graduate School of Convergence Science and Technology, Seoul National University, Seoul, South Korea; 3 Precision Medicine Center, Seoul National University Bundang Hospital, Seongnam, South Korea; 4 Department of Genomic Medicine, Seoul National Bundang Hospital, Seongnam, South Korea; 5 Department of Neurology, Inha University Hospital, Inha University College of Medicine, Incheon, South Korea; 6 Department of Neurology, Seoul National University College of Medicine, Seoul, South Korea; 7 Department of Neurology, Dizziness Center, Clinical Neuroscience Center, Seoul National University Bundang Hospital, 173-82 Gumi-ro, Bundang-gu, Seongnam, Gyeonggi-do 13620 South Korea

**Keywords:** Dizziness, Nystagmus, Episodic ataxia, Intellectual disability, Calcium channel

## Abstract

**Background:**

Episodic ataxia type 2 (EA2) results from pathogenic variants in *CACNA1A* that encodes the CaV2.1 P/Q-type calcium channel. The molecular basis of cognitive impairments requires further elucidation in EA2.

**Objective:**

To correlate AI-predicted structural alterations of the CaV2.1 channel with intellectual function observed in patients with EA2.

**Methods:**

Using AlphaFold3, we modeled the wild-type and variant *CACNA1A* proteins. Structural similarity between the wild-type and variant proteins was quantified using the Template Modeling (TM) score. To assess degree of truncation, the relative amino acid length ratio (AA%) was also calculated. These protein-level metrics were then compared with the standardized intellectual indices in 13 patients with EA2.

**Results:**

The TM scores ranged from 0.624 to 0.838, and showed a strong correlation with most intellectual indices, including the full-scale IQ (FSIQ, *r* = 0.722, *p* = 0.005), verbal comprehension index (VCI, *r* = 0.834, *p* < 0.001), perceptual reasoning index (PRI, *r* = 0.624, *p* = 0.023), and working memory index (WMI, *r* = 0.700, *p* = 0.008). The AA% ranged from 50.6% to 100%, and also showed a correlation with VCI (*r* = 0.566, *p* = 0.044) and WMI (*r* = 0.649, *p* = 0.016), but less consistently when compared to the TM score.

**Conclusions:**

Structural preservation of CaV2.1 correlates more strongly with intellectual function in patients with EA2 than protein length, which suggests that structural disruption of the CaV2.1 channel may contribute to cognitive impairments in EA2. AI-based protein modeling is a valuable tool for linking genotype to phenotype, particularly in channelopathies with diverse clinical presentation.

## Introduction

Episodic ataxia type 2 (EA2) is an autosomal dominant channelopathy characterized by recurrent episodes of vertigo, gait ataxia, and dysarthria, often triggered by exertion or emotional stress [9, 10]. EA2 results from pathogenic variants in *CACNA1A*, encoding the α1A subunit of the P/Q-type voltage-gated calcium channel (CaV2.1) that plays a critical role in neurotransmitter release at cerebellar and cortical synapses [17, 19]. Defects in *CACNA1A* cause impaired calcium influx and neuronal excitability that lead to attacks of ataxia and interictal cerebellar signs in EA2 [19, 25].

In addition to intermittent ataxia and cerebellar ocular motor findings, recent reports have described cognitive impairment or intellectual disability in patients with EA2 [13, 14]. Such findings suggest that *CACNA1A*-related neuronal dysfunction may extend beyond cerebellar motor circuits to involve cortico-cerebellar networks relevant to cognitive processing. However, the molecular mechanisms linking *CACNA1A* variants to intellectual disability require further elucidation.

Different classes of *CACNA1A* mutations—missense, nonsense, splice-site, or frameshift—can produce diverse structural changes in the CaV2.1 channel, depending on the affected domains such as the voltage-sensing S4 segments or the pore-forming S5–S6 loops [4, 19]. Characterizing the structural impact of these mutations may explain the phenotypic heterogeneity observed in EA2 patients. With recent advances in AI-based protein structure prediction, particularly AlphaFold3 (alphafoldserver.com, Google DeepMind), it is now possible to estimate mutation-induced structural alterations of proteins, including changes in length, folding confidence, and structural similarity to the wild-type protein [1]. These protein-level metrics may provide quantitative indicators of functional impairments, potentially bridging the gap between genetic variations and clinical phenotypes in EA2.

Therefore, we aimed to determine whether the protein-level metrics of *CACNA1A* variants, which were estimated using AlphaFold3, correlate with the degree of intellectual disability in patients with EA2. By linking genetic alterations to predicted protein-level disruptions and clinical phenotypes, this study aims to provide insight into the molecular mechanisms underlying cognitive impairments in EA2 and to define the protein structure-based indicators for early diagnosis and prognosis of EA2.

## Methods

### Participants

We studied the same cohort of patients with EA2 (13 patients from nine families) that was previously reported in our clinical study of cognitive function and intellectual disability in EA2 (Table 1) [14]. As described in that study, genetic testing was performed by direct sequencing in eight patients and by next-generation sequencing (NGS) in five patients. All patients carried pathogenic or likely pathogenic variants in *CACNA1A*.

**Table 1:** Intellectual function using Wechsler Adult Intelligence Scale and clinical characteristics of patients

Case ID	Sex	Age	FSIQ	VCI	PRI	WMI	PSI	Age at onset	Attack frequency (per month)	Duration (hours)	Triggering factors	Interictal ocular motor findings	Response to acetazolamide	Other clinical features
1	M	52	101	90	103	109	110	10	1	12	Exercise	GEN, DBN, pHIT	+	
2	M	18	99	100	94	125	81	5	1	24–36	Exercise, concentration	GEN, DBN, convergence upbeat, pHIT	+	
3	F	23	86	90	103	93	75	9	4	1	Exercise, menstruation	GEN, DBN	+	Tinnitus, earfullness
4	F	47	83	76	96	81	98	10	Almost resolved	0.5	Fatigue	GEN, DBN	+	Migraine
5	M	41	80	77	88	98	81	19	2	2–3	Exercise, fatigue, cold	GEN, DBN, pHIT	+	Febrile seizure
6	F	10	80	86	84	83	92	4	5–6	Several hours	Unknown	GEN, DBN, CPN	+	Febrile seizure
7	F	34	80	83	92	90	78	9	2	24	Exercise	GEN, DBN, increased AC gain during HI	+	Migraine, tonic up-gaze, developmental delay, spasmus nutans
8	M	46	77	88	74	75	97	30	0.5	2	None	GEN, DBN, CPN, impaired SP	+	Headache
9	M	17	67	74	68	78	84	13	4	1	None	GEN, DBN, vertical saccadic slowing	+	
10	F	39	66	70	76	69	84	38	15	1	None	GEN, pHSN, increased AC gain during HI	+	
11	F	59	65	76	70	87	66	10	2	2–3	Stress	GEN, loss of torsional saccades	+	
12	M	17	64	68	76	78	66	1.2	2–3	0.5–1	Exercise, stress, overeating	GEN, DBN	+	
13	F	22	63	72	72	63	78	12	10–15	1	Exercise	GEN, DBN	+	

AC, anterior semicircular canals; CPN, central positional nystagmus; DBN, downbeat nystagmus; FSIQ, Full Scale Intelligence Quotient; GEN, gaze-evoked nystagmus; HI: head-impulse; pHIT, perverted head impulse test; pHSN, perverted head-shaking nystagmus; PRI, Perceptual Reasoning Index; PSI, Processing Speed Index; VCI, Verbal Comprehension Index; WMI, Working Memory Index

All experiments were in accordance with the Declaration of Helsinki, and informed consent was not required because this study was retrospective and did not affect subject care in any way. The Institutional Review Board of Seoul National University Bundang Hospital approved this study (IRB no. B-2406-907-102).

### Sequence data retrieval and annotation

Genomic, transcript, and protein sequence information for the human *CACNA1A* gene was retrieved from the NCBI Reference Sequence (RefSeq) database using the Entrez Programming Utilities (E-utilities), the NCBI API for programmatic data access. The retrieved sequences included the genomic (accession: NG_011569.1), mRNA (accession: NM_001127221.1), and protein sequences (accession: NP_001120693.1). Functional domain information was obtained from the “misc_feature” annotations in the RefSeq record, which describe additional structural or regulatory motifs not categorized as standard coding features.

### Variant protein sequence generation

Genomic positions of *CACNA1A* variants were identified and validated using the GeneBe database (https://genebe.net) [23], which provides comprehensive variant annotations, including genomic coordinates, transcript-level nomenclature, and predicted functional effects. Variant protein sequences were generated by introducing cDNA-level mutations into the wild-type coding sequence (CDS) using custom Python scripts (version 3.8; https://github.com/jinoklee/CACNA1A_Alphafold.git) with the Biopython library (version 1.83). Missense and indel variants were modeled by applying the corresponding nucleotide substitutions, insertions, or deletions to the CDS, and the resulting sequences were translated in silico. For the splicing variant c.4953 + 1G > A (chr19:g.13245181C > T, hg38), SpliceAI (https://spliceailookup.broadinstitute.org; Illumina, Inc.) [8] predicted a donor loss score of 0.93, indicating disruption of the canonical splice donor site. Intron retention was modeled by translating the retained intronic sequence until the first downstream stop codon. The relative amino acid length ratio (AA%), representing the proportion of the predicted variant protein length compared with the full-length wild-type CACNA1A (2261 residues), was calculated for each variant to quantify the degree of truncation. To predict the likelihood of nonsense-mediated mRNA decay (NMD) for each identified variant, we performed variant annotation using the Ensembl Variant Effect Predictor (VEP, https://asia.ensembl.org/info/docs/tools/vep/script/vep_plugins.html; version 115) with the NMD plugin [16]. The NMD plugin classifies variants as NMD-escaping when they meet established criteria, including localization within the last coding exon, within 50 bases upstream of the penultimate exon–exon junction, within the first 100 coding bases of the transcript, or in an intronless transcript. Variants not meeting these criteria were considered predicted to undergo NMD.

### Protein structure prediction

Three-dimensional structures of both wild-type and variant CACNA1A proteins were predicted using the AlphaFold3 server. Full-length amino acid sequences were submitted independently, and predictions were generated using default parameters. The resulting models were downloaded in CIF format for subsequent structural comparison and visualization analyses.

### Structural similarity assessment

Structural similarity between the wild-type and variant CACNA1A protein models was assessed using the Template Modeling (TM-score) algorithm. TM-score values were computed using the TM-score program (https://zhanggroup.org/TM-score), which provides a normalized measure of global structural similarity ranging from 0 to 1. This metric was used to evaluate the structural impact of amino acid substitutions on overall protein architecture.

### Protein structure visualization and annotation

Protein structures were visualized using the PyMOL Molecular Graphics System (version 3.1; Schrödinger, LLC; https://pymol.org). Functional domain annotations derived from the “misc_feature” fields in the RefSeq record were mapped onto the three-dimensional models to highlight key functional regions, including transmembrane segments, structural domains, and regulatory sites.

### Evaluation of intellectual function

Intellectual function was evaluated using WAIS-IV or the WISC-IV [12, 26]. The WAIS-IV and WISC-IV consist of four subtests: Verbal Comprehension Index (VCI), Perceptual Reasoning Index (PRI), Working Memory Index (WMI), Processing Speed Index (PSI). Each derived from standardized core and supplemental subtests, as described previously [26]. The full-scale IQ (FSIQ), the overall intelligence level of an individual, was calculated based on the 10 core subtests [26]. The FSIQ in the WAIS-IV typically has a mean score of 100 and a standard deviation of 15 [26]. Thus, 68% of people score within a standard deviation of the mean (85–115). The WAIS-IV classifies IQ scores into different levels [very superior (130 and above), superior (120–129), high average (110–119), average (90–109), low average (80–89), borderline (70–79), intellectual disability (69 and below)] [26]. The Korean versions of WAIS-IV or WISC-IV were administered via paper-and-pencil or digitally, and it typically took 70–100 min to complete the core subtests [12].

### Statistical analyses

Continuous variables were summarized as mean ± SD. Correlation analyses were performed to examine the relationships between protein structural metrics (TM-score and AA%) and cognitive measures, including the FSIQ and its four index scores—VCI, PRI, WMI, and PSI. Pearson’s correlation coefficients were calculated for normally distributed data, and Spearman’s rank correlation coefficients were used otherwise. To account for familial clustering (13 patients from 9 families), the linear mixed-effects models (LMMs) were fitted with family included as a random intercept. Cognitive indices were specified as dependent variables, and TM-score and AA% were entered as fixed effects. Regression coefficients (*β*), 95% confidence intervals (CI), and two-tailed *p*-values were reported. Given the exploratory nature of the study and the limited sample size, *p*-values were adjusted for multiple comparisons using the false discovery rate (FDR) method. Statistical significance was defined as two-tailed *p* < 0.05. All analyses were performed using R software (version 4.3.3; R Foundation for Statistical Computing, Vienna, Austria).

## Results

### Prediction of protein structures

**Fig. 1:**
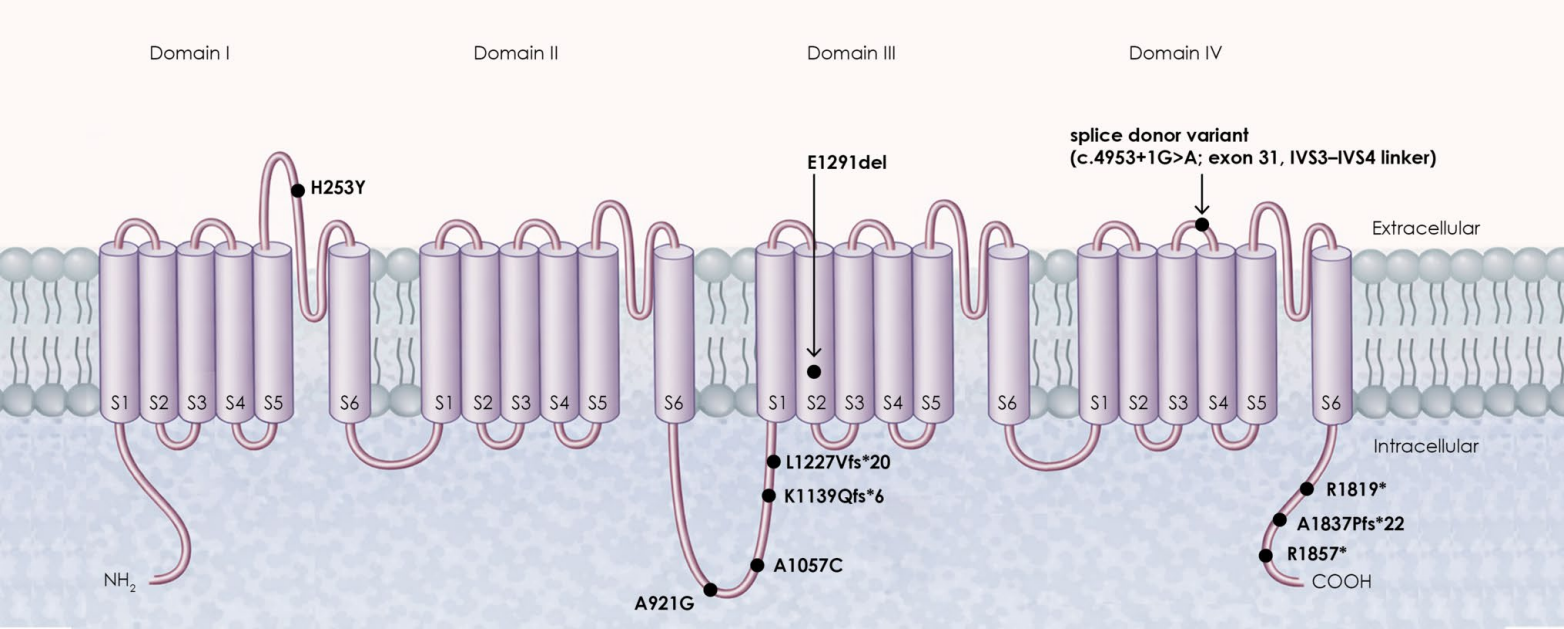
Schematic representation of the Cav2.1 (*CACNA1A*) channel and locations of the pathogenic variants observed in our patients. The Cav2.1 α1A subunit consists of four homologous domains (I–IV), each containing six transmembrane segments (S1–S6) connected by intracellular and extracellular linker regions. The S4 segments function as voltage sensors, and the S5-S6 regions form the pore-forming domain. Black circles indicate the positions of pathogenic variants identified in our patients, including missense variants (p.H253Y, p.A921G, p.A1057C), frameshift variants (p.K1139Qfs*6, p.L1227Vfs*20, p.A1837Pfs*22), nonsense variants (p.R1819*, p.R1857*), an in-frame deletion (p.E1291del), and a splice donor variant (c.4953 + 1G > A) located in the extracellular linker between the segments S3 and S4 of domain IV

The pathogenic variants of *CACNA1A* identified in the patients are summarized in Table 2. The variants were distributed across multiple exons and included stopgain (*n* = 4), missense (*n* = 3), splicing (*n* = 3), frameshift (*n* = 3), and nonframeshift (*n* = 2) mutations. To visualize their spatial distribution, variant sites were mapped onto the schematic topology of the Cav2.1 α1A subunit, which consists of four homologous domains (I–IV) and six transmembrane segments in each domain (Fig. 1).

**Table 2:** Genetic and structural characteristics of *CACNA1A* variants identified in patients with episodic ataxia type 2

Case ID	Family ID	Exonic function	Exon	Coding mutation	Protein mutation	AA%	TM-score	Mean pLDDT	LCR (%)	Predicted NMD
Wild-type	–	–	–	–	100.0	1.000	66.51	34.06	NA	
P1	F1	stopgain	37	c.5569C > T	p.R1857*	82.1	0.816	70.23	26.19	likely
P2	F1	stopgain	37	c.5569C > T	p.R1857*	82.1	0.816	70.23	26.19	likely
P3	F2	nonsynonymous SNV, stopgain	19,37	c.2762C > G, c.5569C > T	p.A921G, p.R1857*	82.1	0.838	70.32	26.72	likely
P4	F3	splicing	31^	c.4953 + 1G > A	unknown	76.8	0.716	66.23	33.22	likely
P5	F4	nonframeshift substitution	23	c.3871_3873delGAG	p.E1291del	100.0	0.741	66.32	33.94	unlikely
P6	F4	nonframeshift substitution	23	c.3871_3873delGAG	p.E1291del	100.0	0.741	66.32	33.94	unlikely
P7	F5	nonsynonymous SNV, frameshift substitution	20,36	c.3169C > T, c.5509delG	p.A1057C, p.A1837Pfs*22	82.1	0.795	69.34	26.93	likely
P8	F6	stopgain	36	c.5455C > T	p.R1819*	80.4	0.780	69.62	27.61	likely
P9	F3	splicing	31^	c.4953 + 1G > A	Unknown	76.8	0.716	66.23	33.22	likely
P10	F7	frameshift substitution	21	c.3679_3681delinsG	p.L1227Vfs*20	55.1	0.624	61.95	40.24	likely
P11	F8	nonsynonymous SNV	5	c.757C > T	p.H253Y	100.0	0.753	66.39	34.06	unlikely
P12	F9	frameshift substitution	20	c.3414dup	p.K1139Qfs*6	50.6	0.712	65.85	35.08	likely
P13	F3	splicing	31^	c.4953 + 1G > A	Unknown	76.8	0.716	66.23	33.32	likely

AA %, relative amino acid length ratio to the wild-type CACNA1A (2261 residues); TM score, Template Modeling score; pLDDT, predicted Local Distance Difference Test; LCR (%), low-confidence residue ratio (proportion of residues with pLDDT < 50)

^intron

**Fig. 2:**
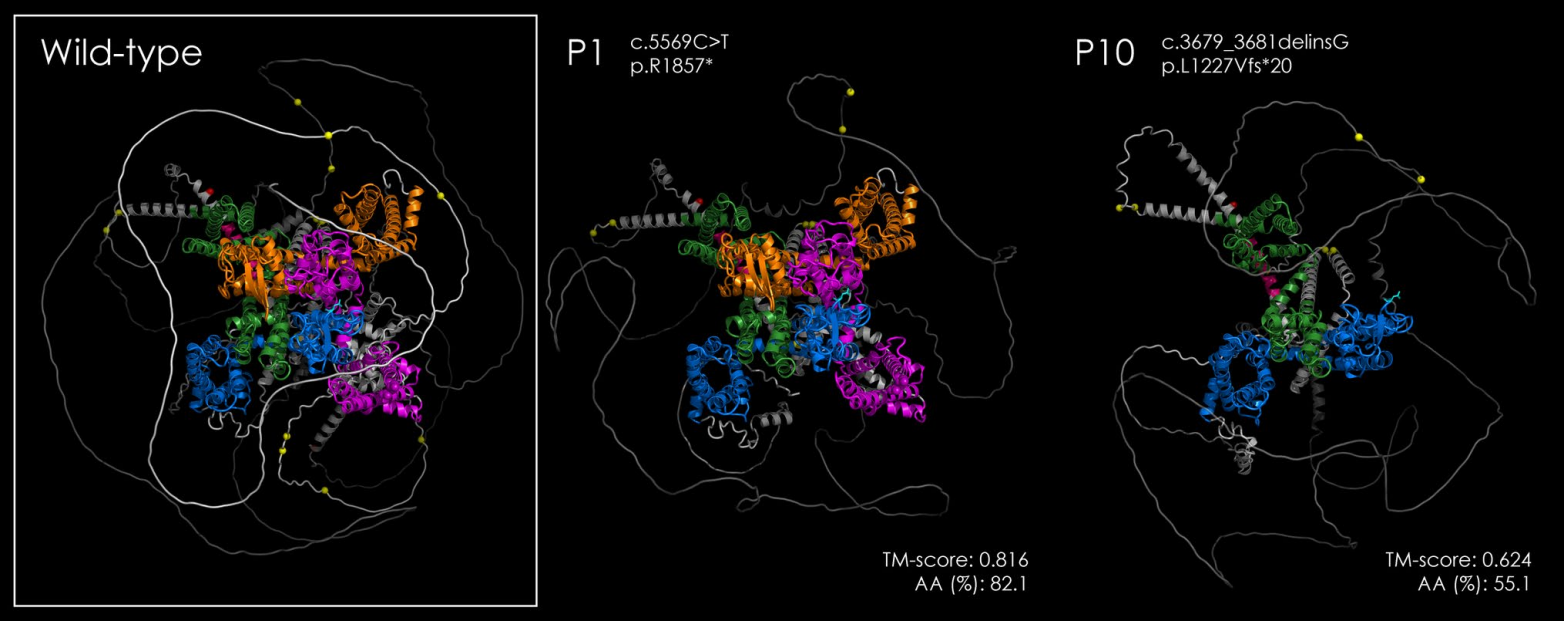
Comparative structural modeling of the Cav2.1 (*CACNA1A*) channel in the wild-type and representative patients. Protein structures were modeled using AlphaFold3 and visualized in PyMOL. The wild-type Cav2.1 channel shows a compact and organized tertiary structure composed of four homologous domains (I–IV), colored as follows: Domain I, blue; Domain II, green; Domain III, orange; and Domain IV, magenta. In Patient 1 (c.5569C > T, p.R1857*), the overall conformation was relatively preserved (TM-score = 0.816; AA% = 82.1), consistent with the patient’s average intellectual performance (FSIQ = 101). In contrast, Patient 10 (c.3679_3681delinsG, p.L1227Vfs*20) showed a severely truncated and disorganized structure (TM-score = 0.624; AA% = 55.1), corresponding to an intellectual disability level. Abbreviations: AA%, relative amino acid length ratio to the wild-type *CACNA1A* (2261 residues); TM-score, template modeling score

Across all patient-derived models, TM-scores ranged from 0.624 to 0.838 and AA% from 50.6% to 100%, which indicates various levels of conformational preservation depending on the mutation type and location (Table 2). Representative examples are shown for patient 1 (p.R1857*, TM-score 0.816, AA% 82.1) and patient 10 (p.L1227Vfs*20, TM-score 0.624, AA% 55.1). Whereas the model of patient 1 showed preservation of most domain architectures, similar to the wild-type, the truncated model of patient 10 exhibited a marked structural disruption and domain loss, consistent with premature termination during protein synthesis (Fig. 2).

Based on NMD plugin classification, truncating and splice-site variants were frequently predicted to undergo NMD. Specifically, the nonsense variants p.R1857* (P1–P3) and p.R1819* (P8), frameshift variants p.L1227Vfs*20 (P10), p.K1139Qfs*6 (P12), and p.A1837Pfs*22 (P7), as well as the splice donor variant c.4953 + 1G > A (P4, P9, P13), were classified as NMD-predicted. In contrast, missense variants (p.H253Y, p.A921G, p.A1057C) and the in-frame deletion p.E1291del (P5–P6) were classified as NMD-escaping according to the plugin criteria. Overall, 76.9% (10/13) of patients were predicted to undergo NMD (Table 2).

### Correlation between protein structural metrics and intellectual function

**Fig. 3:**
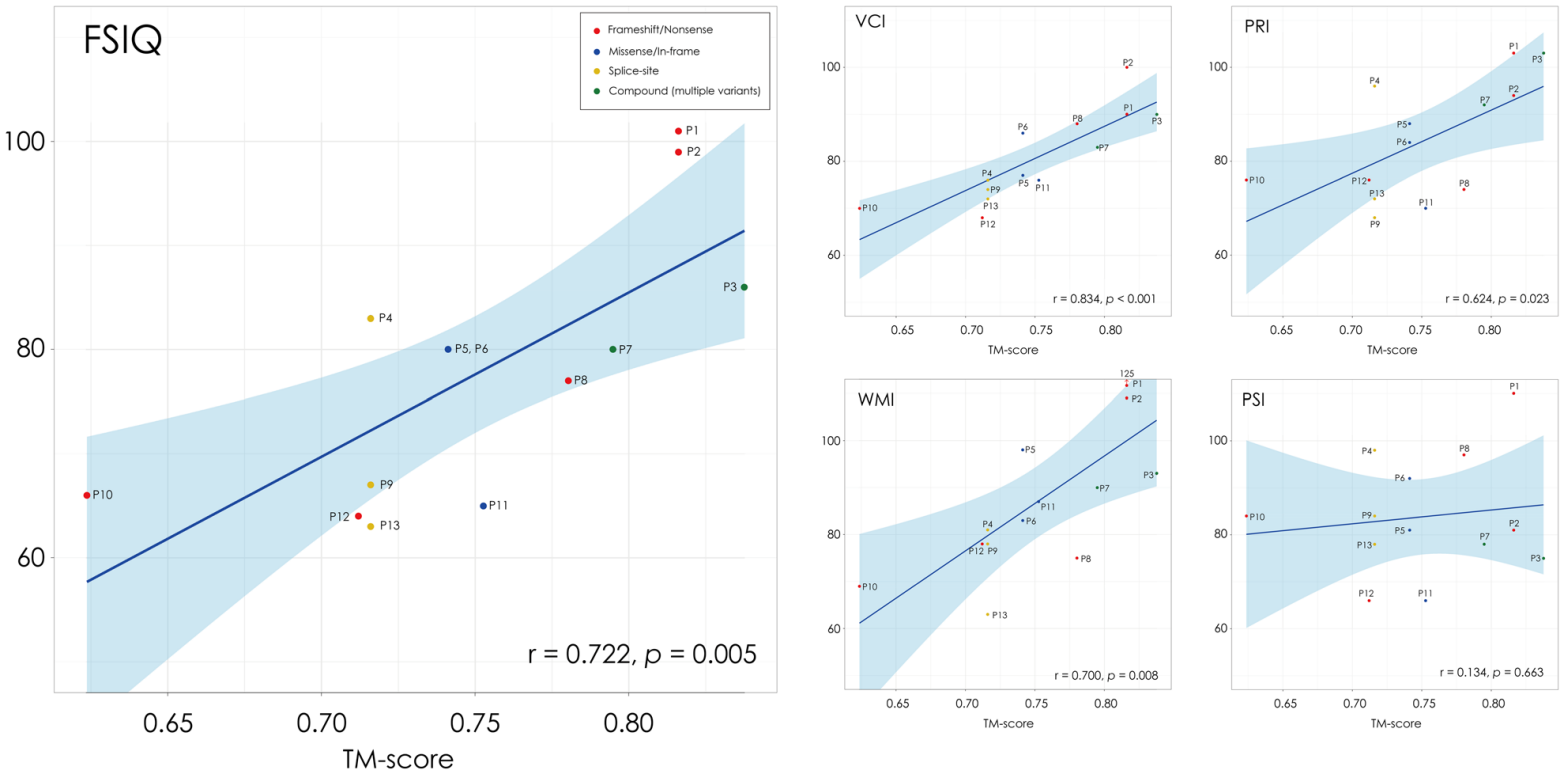
Correlations between structural similarity (TM-score) of proteins and intellectual function. Scatter plots show the correlations between TM-scores and indices of intellectual function, including the full-scale IQ (FSIQ) and its subcomponents: verbal comprehension index (VCI), perceptual reasoning index (PRI), working memory index (WMI), and processing speed index (PSI). Each dot represents a score of individual patient. Shaded areas indicate 95% confidence intervals of the regression line. The TM-scores show a significant positive correlations with FSIQ (*r* = 0.722, *p* = 0.005), VCI (*r* = 0.834, *p* < 0.001), PRI (*r* = 0.624, *p* = 0.023), and WMI (*r* = 0.700, *p* = 0.008) but no significant correlation with PSI (*r* = 0.134, *p* = 0.663). Patients P3 and P7 are categorized as Compound (multiple variants). P3 carried p.A921G and p.R1857*, whereas P7 carried p.A1057C and p.A1837Pfs*22. For these cases, structural metrics were derived from the modeled protein sequence incorporating both variants

The TM-score showed significant positive correlations with several indices of intellectual function (Table 3, Fig. 3) that include the FSIQ (*r* = 0.722, *p* = 0.005), VCI (*r* = 0.834, *p* < 0.001), PRI (*r* = 0.624, *p* = 0.023), and WMI (*r* = 0.700, *p* = 0.008). In contrast, no significant correlation was found with PSI (*r* = 0.134, *p* = 0.663). The relative amino acid length ratio (AA%), which reflects the proportion of predicted amino acid length of the mutant protein to the full-length wild-type sequence, also showed a correlation with VCI (*r* = 0.566, *p* = 0.044) and WMI (*r* = 0.649, *p* = 0.016), but less consistently when compared to the TM-score.

**Table 3:** Correlation between protein structural metrics and intellectual function

Cognitive index	Mean ± SD	TM-score			AA%		
		*r* [95% CI]	*p* value	FDR-adjusted *p-*value	*r*	*p*-value	FDR-adjusted *p* value
FSIQ	77.8 ± 12.2	0.722 [0.28–0.91]	0.005	0.026	0.358	0.230	0.328
VCI	80.8 ± 9.1	0.834 [0.52–0.95]	< 0.001	0.004	0.566	0.044	0.073
PRI	84.3 ± 12.0	0.624 [0.11–0.87]	0.023	0.045	0.179	0.560	0.699
WMI	86.8 ± 16.1	0.700 [0.24–0.90]	0.008	0.026	0.649	0.016	0.041
PSI	83.8 ± 12.2	0.134 [− 0.45–0.64]	0.663	0.737	− 0.102	0.740	0.740

Pearson’s correlation was applied to normally distributed variables (TM-score), whereas Spearman’s rank correlation was used for non-normally distributed variables (AA%). *p* values were adjusted for multiple comparisons using the false discovery rate (FDR) method

AA%, relative amino acid length ratio to the wild-type CACNA1A (2261 residues); FSIQ, Full Scale Intelligence Quotient; PRI, Perceptual Reasoning Index; PSI, Processing Speed Index; TM-score, Template Modeling score; VCI, Verbal Comprehension Index; WMI, Working Memory Index

### Mixed-effects analyses accounting for familial clustering

When using the linear mixed-effects models (Table 4), the TM-score was significantly associated with FSIQ in the univariable model (*β* = 148.18, 95% CI 50.66–245.70, *p* = 0.007; FDR-adjusted *p* = 0.041), whereas AA% was not (*p* = 0.302). In the multivariable model including both predictors, TM-score remained significantly associated with FSIQ (*β* = 151.86, 95% CI 38.20–265.53, *p* = 0.014; FDR-adjusted *p* = 0.041), while AA% was not significant (*p* = 0.883). Variance inflation factors were low for both TM-score and AA% (VIF = 1.27 for each), indicating no meaningful multicollinearity between the predictors.

**Table 4:** Linear mixed-effects models (LMM) account for family clustering

Outcome	Predictor	*β* (95% CI)	*p* value	FDR-adjusted *p-*value
FSIQ	TM-score	148.18 (50.66–245.70)	0.007	0.041
	AA%	25.01 (− 26.08–76.10)	0.302	0.550
	TM-score (adjusted for AA%)	151.86 (38.20–265.53)	0.014	0.041
	AA% (adjusted for TM-score)	− 2.90 (− 46.13–40.33)	0.883	0.883
VCI	TM-score	136.76 (82.46–191.06)	< 0.001	0.002
	AA%	27.46 (− 9.32–64.24)	0.127	0.283
	TM-score (adjusted for AA%)	133.12 (72.05–194.19)	< 0.001	0.004
	AA% (adjusted for TM-score)	3.03 (− 20.44–26.49)	0.785	0.869
PRI	TM-score	134.27 (33.51–235.02)	0.013	0.041
	AA%	14.38 (− 35.64–64.41)	0.540	0.865
	TM-score (adjusted for AA%)	148.54 (36.41–260.68)	0.013	0.041
	AA% (adjusted for TM-score)	− 11.86 (− 54.94–31.22)	0.562	0.865
WMI	TM-score	181.35 (49.92–312.77)	0.011	0.041
	AA%	39.26 (− 24.16–102.69)	0.200	0.400
	TM-score (adjusted for AA%)	171.86 (19.54–324.18)	0.031	0.077
	AA% (adjusted for TM-score)	7.29 (− 50.73–65.31)	0.786	0.869
PSI	TM-score	29.19 (− 100.45–158.83)	0.635	0.869
	AA%	9.77 (− 40.15–59.68)	0.679	0.869
	TM-score (adjusted for AA%)	22.17 (− 123.77–168.12)	0.748	0.869
	AA% (adjusted for TM-score)	5.83 (− 50.25–61.90)	0.826	0.869

β coefficients represent fixed effect. *p* values were adjusted for multiple comparisons using the false discovery rate (FDR) method

AA%, relative amino acid length ratio to the wild-type CACNA1A (2261 residues); FSIQ, Full Scale Intelligence Quotient; PRI, Perceptual Reasoning Index; PSI, Processing Speed Index; TM-score, Template Modeling score; VCI, Verbal Comprehension Index; WMI, Working Memory Index

For domain-specific indices, TM-score was significantly associated with VCI and PRI after FDR correction and showed a marginal association with WMI in the multivariable model. No significant associations were observed between TM-score and PSI. AA% was not significantly associated with any cognitive index in the multivariable analyses.

## Discussion

In this study, we demonstrated that structural similarity between the wild-type and pathogenic variants in CaV2.1 channel, quantified with the TM-score, showed a significant correlation with the degree of intellectual function in patients with EA2. Specifically, higher TM-scores, reflecting better preservation of global protein conformation, were correlated with higher FSIQ and its subindices, including VCI, PRI, and WMI. In contrast, the relative AA%, representing the proportion of predicted protein length to the wild-type sequence, showed weaker and inconsistent relationships. These results suggest that structural integrity rather than protein length per se may more accurately reflect the functional consequences of *CACNA1A* mutations.

The strong association between the TM-score and intellectual measures indicates that the conformational stability of the CaV2.1 channel is crucial for maintaining cognitive integrity. The P/Q-type calcium channel encoded by *CACNA1A* is widely expressed not only in cerebellar Purkinje cells but also in the cerebral cortex, hippocampus, and basal ganglia, where it mediates calcium-dependent neurotransmitter release [24, 27]. Disruption of this channel can therefore impair synaptic transmission in both motor and cognitive networks. Anatomical and functional connectivity studies have demonstrated that cerebellar dysfunction may lead to cognitive deficits by disrupting cerebello-thalamo-cortical circuits, which constitute reciprocal closed-loop pathways between specific cerebellar regions (Crus I/II) and the prefrontal and parietal association cortices [3]. Consistent with this framework, clinical studies of EA2 patients with *CACNA1A* mutations have reported significant cognitive impairments, particularly for executive and verbal functions [13, 14]. Consistent with these findings, our data also showed that patients with greater structural disorganization of the CaV2.1 channel exhibited poorer intellectual performance. This implies that mutation-induced structural disruption of the CaV2.1 channel (misfolding, domain loss, or reduced protein expression) may contribute to impaired calcium-dependent synaptic transmission within cortico-cerebellar circuits involved in higher-order cognition, such as language and reasoning.

In contrast to other cognitive domains, the PSI showed no significant association with structural measures of protein (Table 3, Fig. 3). This finding is consistent with the notion that PSI primarily reflects the degree of white-matter myelination and visuomotor processing speed rather than synaptic efficiency or cortical neurotransmission. Previous studies have reported that processing speed is largely determined by axonal conduction velocity and myelination [5, 18], which are relatively independent of CaV2.1 channel function. Therefore, the dissociation between PSI and other cognitive indices suggests that cognitive impairments in EA2 stem primarily from channel dysfunction within the cortico-cerebellar networks rather than from generalized brain dysfunction.

Our study proposes a conceptual model for linking molecular alterations to neuropsychological outcomes in channelopathies. The use of AlphaFold3-based protein modeling allowed us to quantify the extent of structural disruption for each *CACNA1A* variant and to relate it directly to patient-level cognitive data. Such an approach offers a novel avenue for understanding the genotype–phenotype relationship in rare neurological disorders. Structural similarity metrics such as the TM-score could serve as surrogate markers of pathogenicity and may help predict clinical outcomes even in the absence of electrophysiological or imaging data. Beyond EA2, this methodology may be applicable to other *CACNA1A*-related disorders, including spinocerebellar ataxia type 6 and familial hemiplegic migraine, which also exhibit variable cognitive and behavioral features [19].

The confidence of each predicted model was evaluated using the predicted Local Distance Difference Test (pLDDT), which provides a per-residue or atomic-level confidence score ranging from 0 to 100, with higher values indicating greater reliability of the predicted structure. In this study, the mean pLDDT values of the wild-type and patient models ranged from 61.95 to 70.23 (Table 2), indicating moderate reliability. Nevertheless, this confidence level warrants a cautious interpretation because such models may contain inaccuracies regarding fine structural details. This is particularly relevant for certain regions of the CaV2.1 protein that are known to be intrinsically disordered and thus challenging to model with high precision [7, 11]. However, it should be noted that certain regions of the CaV2.1 protein are inherently difficult to model accurately. The CaV2.1 channel contains long cytoplasmic linkers and termini that are predicted to be intrinsically disordered or highly flexible [4]. As highlighted by recent studies, a substantial fraction of regions with low or very low confidence scores in AlphaFold predictions correspond to intrinsically disordered regions, which reflects conformational heterogeneity rather than model failure [6, 21].

Splice-site variants are known to produce heterogeneous transcript outcomes, including exon skipping, intron retention, or activation of cryptic splice sites [22]. In the present study, structural modeling of the c.4953 + 1G > A variant was performed under an intron retention scenario as a representative consequence of canonical splice donor disruption. However, we acknowledge that alternative splicing outcomes cannot be excluded.

Importantly, NMD prediction using the VEP NMD plugin classified this splice-site variant, as well as most truncating variants, as likely to undergo nonsense-mediated decay. This suggests that the mutant transcripts may be degraded prior to translation, resulting in reduced protein production. In such cases, haploinsufficiency rather than structural misfolding of an aberrant protein may represent the predominant pathogenic mechanism [15, 20].

Overall, our findings indicate that distinct molecular mechanisms may underlie different variant classes in *CACNA1A*. Missense and in-frame variants are more likely to affect channel structure or function directly (NMD-escaping), whereas truncating and splice-site variants may primarily act through reduced protein expression due to NMD. Taken together, our findings suggest that distinct pathogenic mechanisms, including haploinsufficiency resulting from NMD and structural perturbation of the CaV2.1 channel, may coexist depending on transcript fate.

Nevertheless, several limitations should be noted. First, the sample size was relatively small, reflecting the rarity of EA2, and thus the correlations observed here should be interpreted with caution. Replication in larger and more diverse cohorts will be necessary to confirm these findings. Second, the structural predictions were generated from AI-based in silico modeling and have not been experimentally validated. Although AlphaFold3 achieves remarkable accuracy across many proteins, the model primarily predicts a single static conformation and may produce hallucinated or stereochemical errors [1, 2]. It also has inherent limitations in capturing dynamic processes such as channel gating, post-translational modifications, or interactions with auxiliary subunits that are critical for channel function [1, 2]. Third, given the observational design, our findings do not establish a causal relationship between predicted structural alterations and cognitive outcomes. Although an association was observed, broader neurodevelopmental factors, cerebello-cortical network involvement, overall disease burden (including migraine or epilepsy), and potential medication effects may also contribute to cognitive performance and cannot be excluded. Accordingly, our results support a link between predicted structural alterations and cognitive performance; however, the precise pathophysiological mechanisms warrant further functional and clinical investigation.

In conclusion, our findings suggest that the degree of structural preservation of the CaV2.1 channel, rather than the extent of truncation, is closely associated with intellectual function in EA2. By integrating AI-driven protein modeling with clinical neuropsychological assessment, this study provides preliminary evidence that structural integrity of *CACNA1A* may serve as a molecular correlate of cognitive capacity. This protein-level approach offers a promising framework for elucidating the mechanisms of intellectual disability in channelopathies and may eventually contribute to individualized prognosis and targeted interventions for patients with *CACNA1A* mutations.

## Data Availability

Anonymized data will be shared by request from any qualified investigator.
